# Reactive oxygen species in status epilepticus

**DOI:** 10.1002/epi4.12691

**Published:** 2023-01-28

**Authors:** Matthew C Walker

**Affiliations:** ^1^ Department of Clinical and Experimental Epilepsy UCL Queen Square Institute of Neurology London UK

**Keywords:** excitotoxicity, free radicals, mitochondria, reactive oxygen species, status epilepticus

## Abstract

It has long been recognized that status epilepticus can cause considerable neuronal damage, and this has become one of its defining features. The mechanisms underlying this damage are less clear. Excessive activation of NMDA receptors results in large rises in internal calcium, which eventually lead to neuronal death. Between NMDA receptor activation and neuronal death are a number of intermediary steps, key among which is the generation of free radicals and reactive oxygen and nitrogen species. Although it has long been thought that mitochondria are the primary source for reactive oxygen species, more recent evidence has pointed to a prominent role of nicotinamide adenine dinucleotide phosphate (NADPH) oxidase, an enzyme localized in cell membranes. There is burgeoning in vivo and in vitro evidence that therapies that target the production or removal of reactive oxygen species are not only effective neuroprotectants following status epilepticus, but also potently antiepileptogenic. Moreover, combining therapies targeted at inhibiting NADPH oxidase and at increasing endogenous antioxidants seems to offer the greatest benefits.

**Key Point**

- Reactive oxygen species generation is a key intermediary step between NMDA receptor activation and neuronal death in status epilepticus.
- Reactive oxygen species generation in status epilepticus largely occurs through activation of NADPH oxidase.
- Targeting reactive oxygen species in status epilepticus provides an effect approach to preventing neuronal death and epileptogenesis.

## 1 INTRODUCTION

Throughout his career, Claude Wasterlain has been at the forefront of addressing clinically relevant and important questions through experimental science and the judicious use of appropriate animal models. Early in his career, he recognized the potentially devastating effects of status epilepticus and that it was likely that it was the seizure activity rather than the associated hypoxia/hypotension that caused the damage. Fifty years ago, he observed “Our animals are paralyzed and ventilated with oxygen and seizures are followed by EEG. This prevents both the initial apnea and competition for the available oxygen between brain and muscle, and insures that the modifications that we observe are the result of epileptic seizures and not simply that of cerebral anoxia. Using this model, we have found that SE is accompanied by severe changes in the brain's protein‐synthesizing machinery…”^1^ A year later, this concept was developed further in a set of seminal papers from Brian Meldrum, and colleagues.^2^, ^3^ These studies demonstrated that, although a certain amount of neuronal damage is secondary to hypoxia, hypoglycemia, and hypotension that occur during status epilepticus, a large proportion of the damage is independent of these factors. Further work established the central role of calcium influx into neurons during status epilepticus and led to the concept of excitotoxicity in which the presence of epileptic activity mediates neuronal death through the excessive activation of glutamate receptors, resulting in large influxes of calcium through primarily NMDA receptors, but also through AMPA receptors lacking the GluA2 subunit, leading to a cascade of reactions culminating in cell death.^4^, ^5^, ^6^, ^7^, ^8^ However, calcium entry is not the only initiator of this cascade, but there is also growing evidence that energy failure, with ATP depletion and mitochondrial failure, plays a central role.^9^

**FIGURE 1:**
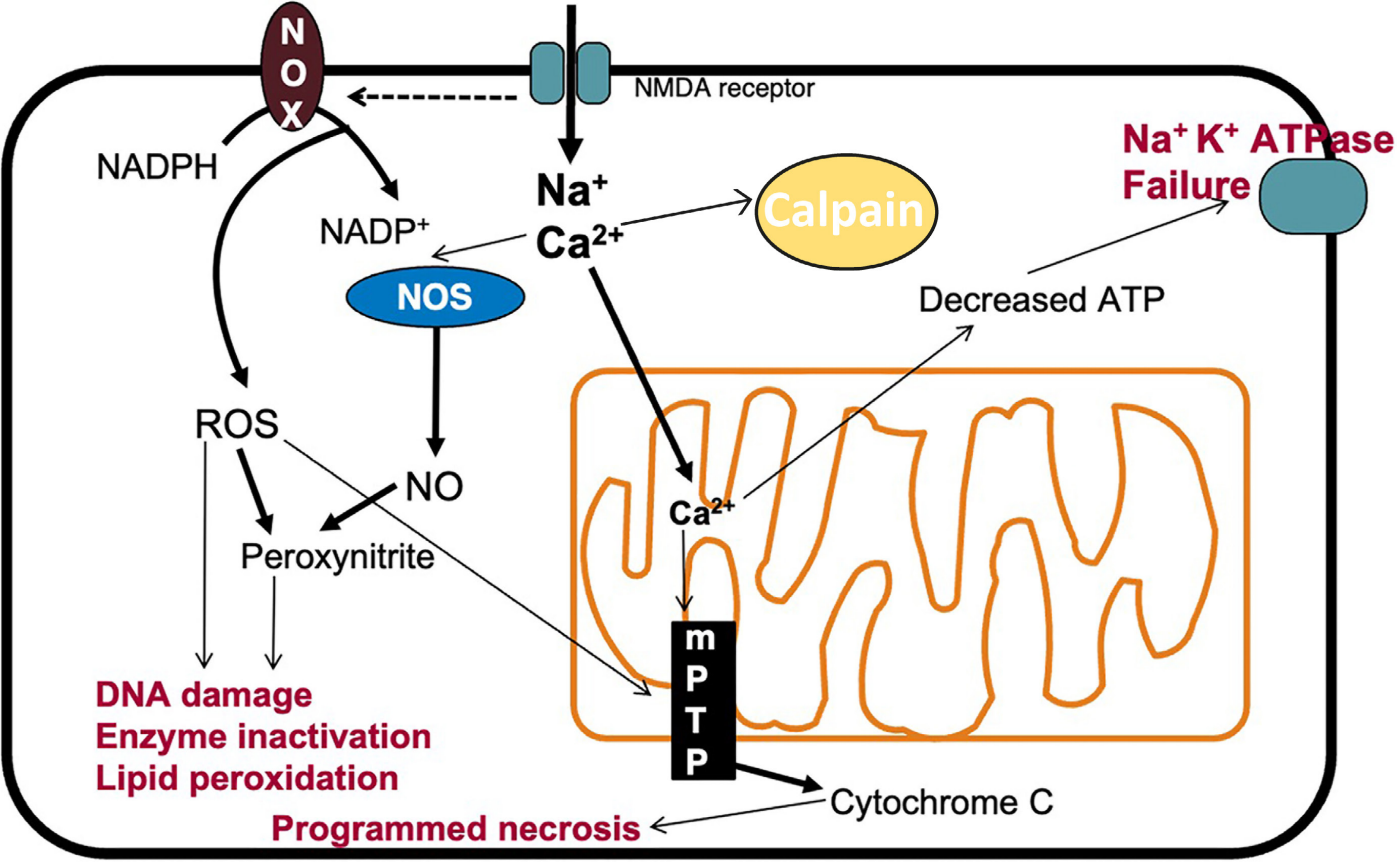
Mechanisms leading to neuronal death following activation of NMDA receptors. NMDA receptor activation and calcium entry activate several enzymes including calpains and nitric oxide synthase (NOS). NADPH oxidase (NOX) is activated by calcium entry but also by nonionotropic effects of NMDA receptor activation. Reactive oxygen species and nitric oxide form peroxynitrite, which is toxic to DNA, proteins, and lipids. Calcium from the cytosol is taken up by the mitochondria, and excessive mitochondrial calcium load results in decreased ATP production, energy failure, and failure to maintain cellular ionic gradients. Mitochondrial calcium accumulation and reactive oxygen species contribute to the formation of the mitochondrial permeability transition pore (mPTP), which further disrupts mitochondrial function, but also permits cytochrome c into the cytosol where it can activate programmed necrosis (From [^12^])

How is it that the influx of calcium and energy failure during status epilepticus lead to cell death? Mechanisms of cell death had conventionally been divided histopathologically into “apoptotic” and “necrotic,” where necrosis is characterized by swelling of the cells and organelles (perceived as a passive process), while apoptosis is characterized by nuclear and cytoplasmic condensation with relative preservation of the organelles and mitochondria (perceived as an active, “programmed” process).^10^ However, the distinction between these two putative forms of cell death is not clear‐cut or even necessarily useful, and it has been proposed to abandon these terms in favor of passive and active cell death.^11^ Indeed, Claude Wasterlain and colleagues identified that in status epilepticus, the predominant form of cell death histologically is necrotic cell death, but that there is a series of steps with release of cytochrome c from mitochondria followed by caspase activation (similar to that observed during “apoptosis”) that led to cell death and so coined the term, programmed necrosis.^12^, ^13^ We and others have tried to fill in the gaps between calcium entry, mitochondrial failure, and programmed necrosis, and we propose that one critical step is the production of reactive oxygen and nitrogen species. Moreover, programmed necrosis is one component of a set of cascades resulting from reactive oxygen species generation that result in energy failure, failure of the sodium‐potassium ATPase and the generation of peroxynitrite that contribute to neuronal death (Figure 1).^14^

## 2 REACTIVE OXYGEN SPECIES GENERATION IN STATUS EPILEPTICUS

**FIGURE 2:**
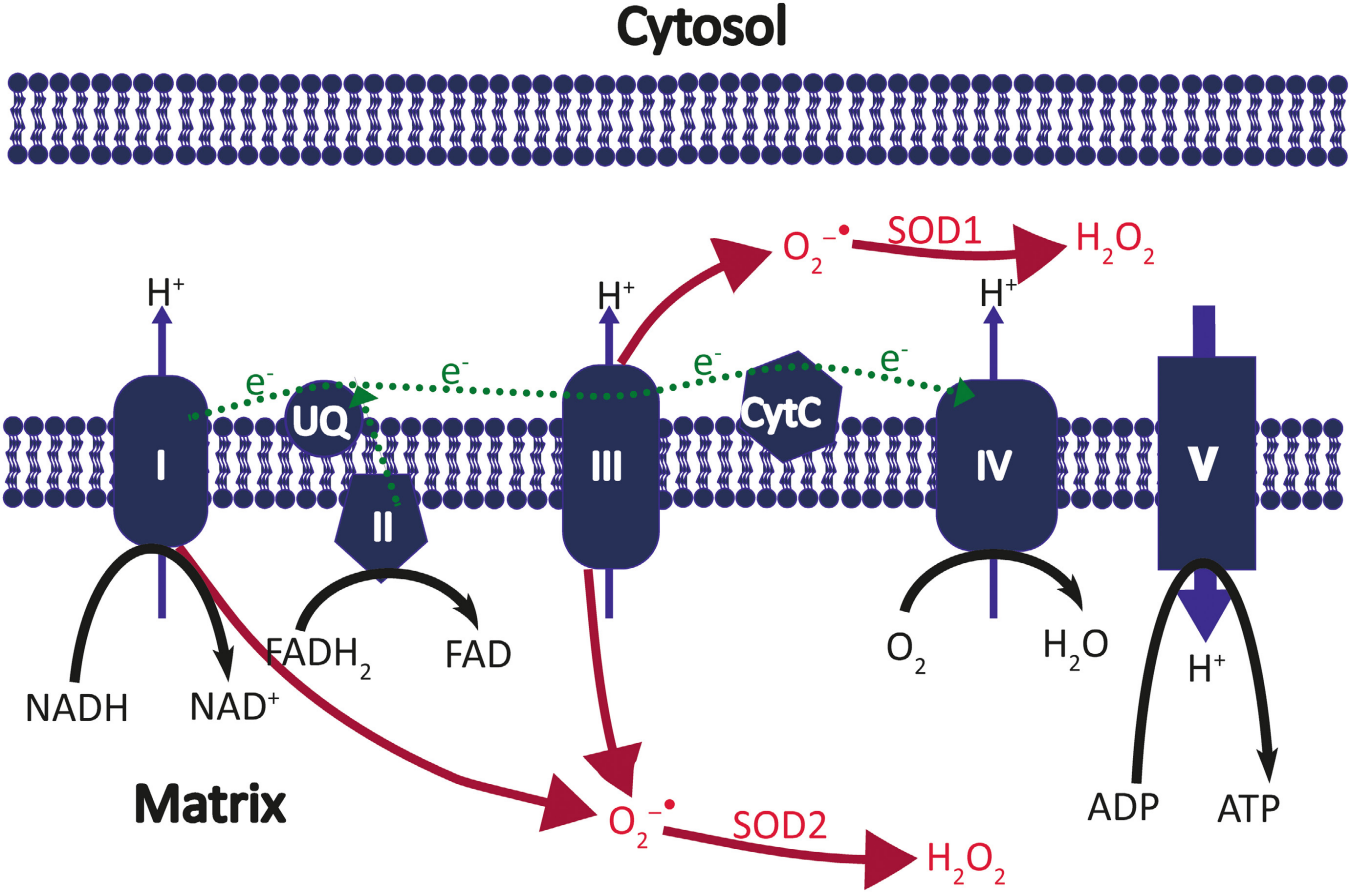
The mitochondria electron transport chain generates superoxide and hydrogen peroxide. This is a simplified diagram of the electron transport chain on the inner mitochondrial membrane, consisting of four complexes (I–IV), ubiquinone (UQ), and cytochrome C (CytC). The green dotted line shows the direction of electron (e^−^) flow along the chain. Energy from the electron transport is used to pump protons (H^+^) across the mitochondrial membrane. The proton gradient is used by Complex V (ATP synthase) to generate ATP. Leakage of electrons from the complexes I and III generates superoxide (O_2_^−•^), which is then converted by superoxide dismutases (SOD1 and SOD2) to hydrogen peroxide (H_2_O_2_)

Although reactive oxygen species and free radicals are often used synonymously, they are different but overlapping entities. Free radicals are defined as molecules that contain at least one unpaired electron in an atomic orbit. Their biological importance is that they are particularly reactive, usually “taking” electrons from other molecules, that is, acting as oxidants. Free radicals can be formed by electron transfer, either a molecule loses an electron (oxidation) or gains an electron (reduction), or through the equal splitting of a covalent bond (homolytic fission).^15^ In biological systems, electron transfer is the mechanism by which free radicals are generated. Mitochondria generate adenosine triphosphate (ATP) through electron transfer and “leakage” of electrons during the mitochondrial electron transfer process can result in reduction of O_2_ to O_2_^−•^ (superoxide; Figure 2); this is a common biological source of free radicals.^16^ Superoxide is a not a particularly damaging free radical and may have a physiological role in several biological processes.

Reactive oxygen species (ROS) refer not only to oxygen free radicals but also to oxygen‐containing molecules that can generate free radicals. Thus, superoxide can react with water to form H_2_O_2_ (hydrogen peroxide; Figure 2), which is not a free radical but is an ROS as it can react with Fe^2+^ or superoxide to form OH^−^ and OH^•^ (the hydroxyl radical). The hydroxyl radical is a particularly reactive ROS and rapidly oxides numerous molecules, inevitably causing cell damage. ROS can also react with nitric oxide to form reactive nitrogen species. One of the most prevalent is peroxynitrite, which is formed though the reaction of nitric oxide, generated by nitric oxide synthase, with superoxide (Figure 1).^17^ Peroxynitrite is particularly toxic for cells, possibly through the formation of OH^•^ and NO_2_^•^. It is responsible for lipid peroxidation, and DNA and protein degradation. It may also be a major driver for mitochondrial failure, and it triggers the release of mitochondrial proapoptotic factors such as cytochrome‐c.^17^ Thus, peroxynitrite can result in programmed necrosis, but also has more far‐reaching effects on cellular function. Hydrogen peroxide and peroxynitrite in low concentrations have important biological roles, and, given their toxicity, their concentrations are closely regulated through free radical scavengers, in particular, catalase and glutathione peroxidase. Indeed, excessive peroxynitrite and hydrogen peroxide production and failure of protective mechanisms, through, for example, glutathione depletion, have been proposed to be a major cause of neuronal death in many neurodegenerative conditions.^18^ There is substantial evidence of an increase in peroxynitrite production in status epilepticus,^19^ and so increases in peroxynitrite production could be a major driver for status‐epilepticus‐associated cell death.

What is it, though, that increases peroxynitrite production? There are two arms to this question, as there needs to be an increase in both nitric oxide formation and superoxide production. Nitric oxide production via neuronal nitric oxide synthase, a calcium/calmodulin‐dependent enzyme, is directly coupled to the influx of calcium through NMDA receptors.^20^, ^21^ However, the generation of superoxide in response to NMDA receptor activation is more complex as there are multiple possible sources. Complexes I and III are the primary sites of ROS production in mitochondria through “electron leak” from the electron transport chain (Figure 2).^22^ During seizure activity, complex III has been considered the main contributor to superoxide production.^23^ However, prolonged seizure activity, such as occurs during status epilepticus, results in mitochondrial depolarization, mitochondrial dysfunction, and energy failure.^24^, ^25^ Therefore, although mitochondria are an important source of ROS during brief seizures, as seizure activity continues, their role in generating ROS diminishes. Using robust protocols for real‐time in vitro and ex vivo monitoring of ROS production during hyperexcitability, we were able to establish that mitochondria are not the main source of ROS during prolonged seizure activity.^26^, ^27^ In contrast, mitochondria are likely a prominent target of ROS‐induced damage during prolonged seizure activity with ROS directly affecting mitochondrial function, inhibiting mitochondrial complex 1 activity, decreasing mitochondrial membrane potential, and inhibiting ATP production.^26^, ^28^, ^29^ Multiple mechanisms including mitochondrial dysfunction, peroxynitrite production, and calcium loading lead eventually to opening of the permeability transition pore,^30^ resulting, as detailed earlier, in programmed necrosis.^12^

Rather than mitochondria, we have proposed that NADPH oxidase is the major source of ROS during status epilepticus.^26^ NADPH oxidases are membrane‐bound enzyme complexes that generate ROS and play an important role in immune defenses.^31^ In the brain, NADPH oxidase has been shown to be activated following NMDA receptor activation and to be the main source of NMDA receptor‐associated cytosolic superoxide production.^32^ Superoxide is relatively short‐lived, and so cytosolic production should facilitate the formation of peroxynitrite through reacting with cytosolic nitric oxide (Figure 1). In addition, the localization of NADPH oxidase to the cell membrane facilitates extracellular release of superoxide and spread to neighboring neurons.^33^ In an in vitro model of prolonged seizure‐like activity, we showed that we could prevent the generation of ROS by inhibiting NADPH oxidase, supporting the prominent role of NADPH oxidase. The generation of ROS was dependent on NMDA receptor activation, but was not significantly affected by removing extracellular calcium.^26^ This supports the hypothesis that NMDA receptor activation can stimulate NAPH oxidase activity independent from calcium entry, possibly through a confirmational change of the receptor^26^, ^33^; indeed, it has been proposed that NMDA receptors can have both ionotropic and nonionotropic roles.^33^

## 3 NEUROPROTECTIVE ROLE OF ANTIOXIDANT STRATEGIES

The observations that free radical generation occurs during status epilepticus and that there are key roles of peroxynitrite and ROS in mitochondrial dysfunction and neuronal death (Figure 1) lead to the supposition that antioxidants should have a neuroprotective effect. There have been reports of the successful use of antioxidants, such as α‐tocopherol/vitamin E in status epilepticus, reducing neuronal injury and inflammation.^34^, ^35^, ^36^ However, many of the studies of antioxidants have mixed results and are confounded by timing of administration, blood‐brain barrier penetration, doses used, and coadministration with other compounds.^37^ In addition, the translational potential of many of the studies is low, as the antioxidant is given prior to the induction of status epilepticus. We have focused on two specific strategies: first increasing antioxidant defenses and second, inhibiting NADPH oxidase. Moreover, we have used these strategies after status epilepticus is established and over a limited period.^27^, ^37^, ^38^, ^39^ In particular, we have increased endogenous antioxidant defenses through activation of nuclear factor erythroid 2‐related factor 2 (Nrf2).^37^, ^38^, ^39^ Nrf2 is a transcription factor that is regulated by Kelch‐like ECH‐associated protein 1 (KEAP 1).^40^ Nrf2 target proteins include not only antioxidant enzymes but also antiinflammatory and metabolic enzymes.^41^ ROS inactivate KEAP1, leading to Nrf2 accumulation and translocation to the nucleus.^42^ Nrf2 increases after status epilepticus,^43^ probably in response to increased ROS production. Genetically induced overexpression of Nrf2 neuroprotects, reduces inflammation, and decreases subsequent seizure frequency following status epilepticus in mice.^43^

**FIGURE 3:**
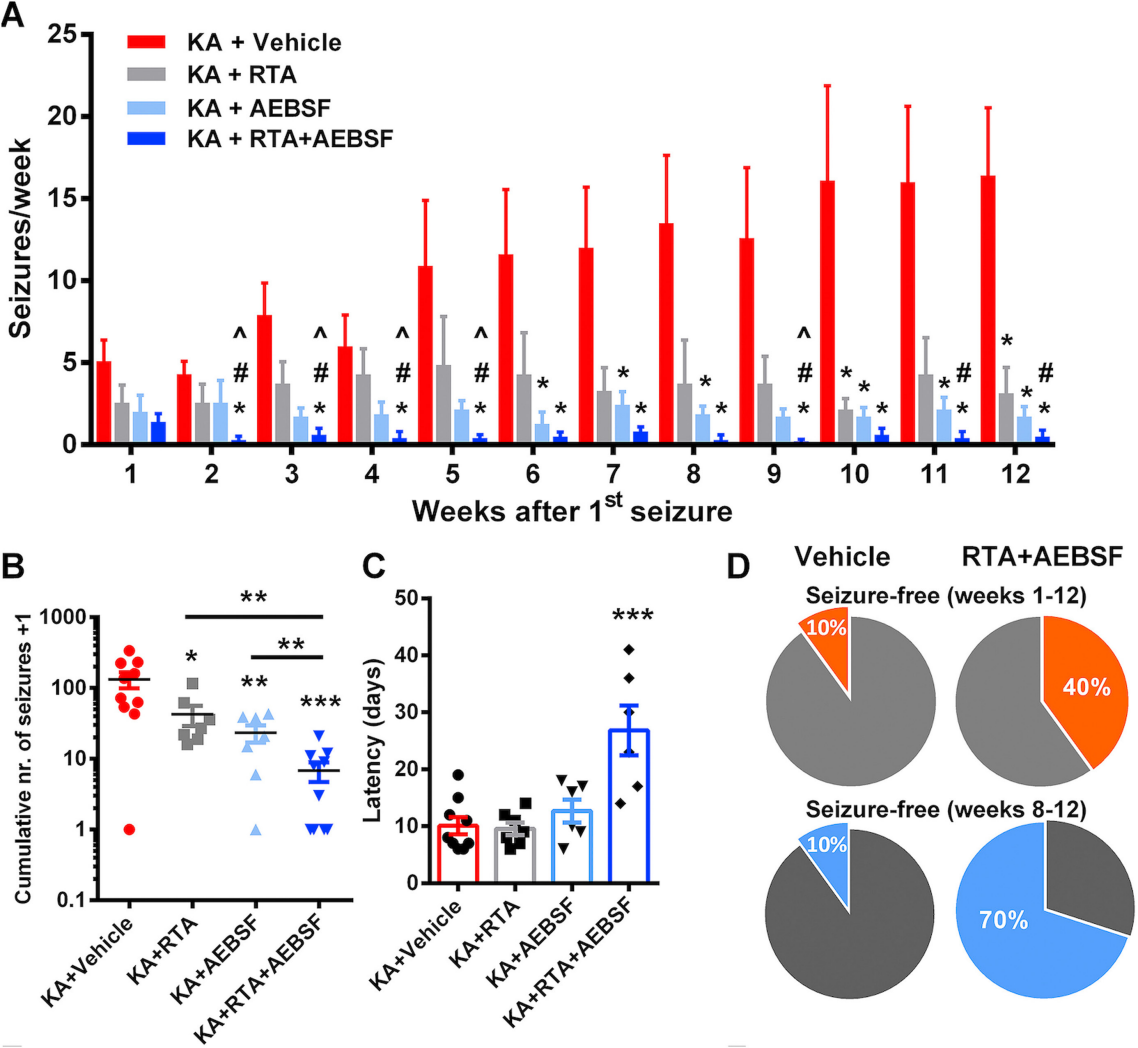
Combination of NADPH oxidase (NOX) inhibition and Nrf2 activation suppresses the development of epilepsy following status epilepticus in rats. A, Bar charts of seizure frequency (seizures\week; mean ± SEM) of animals following KA‐induced status epilepticus (2 h), treated immediately after termination of SE (Diazepam 5 mg/kg) with single administration of either vehicle (10% DMSO/saline; n = 10), RTA 408 25 mg/kg (n = 7), AEBSF 50 mg/kg (n = 7) or RTA 408 25 mg/kg, and AEBSF 50 mg/kg (n = 10). *F*_(3,300)_ = 8.005, *P* < 0.001 by generalized log‐linear mixed model followed by sequential Bonferroni post hoc test. *P < 0.05, vs. Vehicle; #*P* < 0.05, RTA 408 + AEBSF vs. RTA 408; ^*P* < 0.05, RTA 408 + AEBSF vs. AEBSF. B, Cumulative number of seizures of animals in A. Data are plotted on a logarithmic scale after incrementing each total seizure count by one to avoid zero values. *P < 0.05, **P < 0.01, ***P < 0.001, Student's *t*‐test (C) Combination therapy increases the latent period, *F*_(3,24)_ = 11.197, *P* < 0.001, by one‐way ANOVA followed by Tukey's post hoc, ***P < 0.001 vs. KA + Vehicle group. D, The pie charts illustrate percentage of animals seizure‐free for the whole study and for the last 5 weeks following KA‐induced status epilepticus treated with either vehicle (left) or with combination of RTA 408 (25 mg/kg) and AEBSF (50 mg/kg) (right) (From Ref. [^33^])

Nrf2 can be activated by sulforaphane, a naturally occurring substance found in broccoli. Sulforaphane in combination with the antioxidant n‐acetylcysteine given after status epilepticus, induced by electrical stimulation, in rats decreased neuronal damage, subsequent seizures, and cognitive deficits.^38^ This combination was used because the antioxidant effects of Nrf2 activation are not immediate but are long‐lasting, while n‐acetylcysteine has an immediate but not an enduring effect. Sulforaphane has, however, poor penetration of the CNS, so we tested a CNS penetrant, more potent and specific inhibitor of KEAP1, RTA‐408. RTA‐408 given once daily over 3 days following kainic acid‐induced status epilepticus in rats resulted in almost complete neuroprotection in the hippocampus and reduced the occurrence of subsequent spontaneous seizures by more than 90% despite the probable delay in its maximal effect.^39^ We also demonstrated that inhibiting NADPH oxidase with AEBSF, an irreversible serine protease inhibitor, which undoubtedly has some other off‐target effects, similarly neuroprotects and prevents the development of epilepsy following status epilepticus in rats (Figure 3).^27^, ^37^ However, more impressively combining a single dose of the Nrf2 activator (increasing antioxidant defenses) and a single dose of an NADPH oxidase inhibitor (decreasing ROS production) following status epilepticus was neuroprotectant and prevented the later development of epilepsy in 70% of animals (Figure 3). The potency of this combination probably derives from the immediate action of NADPH oxidase inhibition and the more enduring action of Nrf2 activation.

## 4 FREE RADICALS ARE JUST ONE PART OF THE JIGSAW

It is a mistake to attribute the consequences of status epilepticus to one pathway or mechanism. Along with ROS generation, numerous other key and interrelated processes occur including inflammation,^44^, ^45^ release of adenosine^46^ and activation of purinergic receptors, and leakage of the blood‐brain barrier.^47^ Moreover, there are alterations in gene expression that occur through changes in transcription factors, microRNA expression, and mRNA polyadenylation.^48^, ^49^, ^50^ The concept of multiple targets gives rise to the idea of targeted polytherapy but also emphasizes the importance of biomarkers to determine which therapies should be given and when.^51^, ^52^ Although we and others have shown a key role for ROS and the potential for targeting these for neuroprotection and antiepileptogenesis, this needs to be seen in the context of the large body of promising work investigating other targets. Nevertheless, through the pioneering work of Wasterlain and others, we are approaching a point where we have potential therapies that can prevent the consequences of status epilepticus and the development of epilepsy, and the challenge is now to translate these into clinical practice.

## CONFLICT OF INTEREST

MW has no conflict of interest to disclose.

## ETHICAL APPROVAL

I confirm that I have read the Journal's position on issues involved in ethical publication and affirm that this report is consistent with those guidelines.
